# Evaluation of Insurance Coverage and Cancer Stage at Diagnosis Among Low-Income Adults With Renal Cell Carcinoma After Passage of the Patient Protection and Affordable Care Act

**DOI:** 10.1001/jamanetworkopen.2021.16267

**Published:** 2021-07-16

**Authors:** Juan F. Javier-DesLoges, Julia Yuan, Shady Soliman, Kevin Hakimi, Margaret F. Meagher, Fady Ghali, Walter Hsiang, Devin N. Patel, Simon P. Kim, James D. Murphy, J. Kellogg Parsons, Ithaar H. Derweesh

**Affiliations:** 1 Department of Urology, University of California, San Diego, School of Medicine, La Jolla; 2 University of California, San Diego, School of Medicine, La Jolla; 3 Department of Urology, Yale University School of Medicine, New Haven, Connecticut; 4 Department of Urology, University of Colorado Anschutz School of Medicine, Denver; 5 Department of Radiation Medicine and Applied Sciences, University of California, San Diego, School of Medicine, La Jolla

## Abstract

**Importance:**

The association of the Patient Protection and Affordable Care Act (ACA) with insurance status and cancer stage at diagnosis among patients with renal cell carcinoma (RCC) is unknown.

**Objective:**

To test the hypothesis that the ACA may be associated with increased access to care through expansion of insurance, which may vary based on income.

**Design, Setting, and Participants:**

This retrospective cohort analysis included patients diagnosed with RCC from January 1, 2010, to December 31, 2016, in the National Cancer Database. Data were analyzed from July 1 to December 31, 2020. The periods from 2010 to 2013 and from 2014 to 2016 were defined as pre- and post-ACA implementation, respectively. Patients were categorized as living in a Medicaid expansion state or not.

**Exposures:**

Implementation of the ACA.

**Main Outcomes and Measures:**

The absolute percentage change (APC) of insurance coverage was calculated before and after ACA implementation in expansion and nonexpansion states. Secondary outcomes included change in stage at diagnosis, difference in the rate of insurance change, and change in localized disease between expansion and nonexpansion states. Adjusted difference-in-difference modeling was performed.

**Results:**

The cohort included 78 099 patients (64.7% male and 35.3% female; mean [SD] age, 54.66 [6.46] years), of whom 21.2% had low, 46.2% had middle, and 32.6% had high incomes. After ACA implementation, expansion states had a lower proportion of uninsured patients (adjusted difference-in-difference, −1.14% [95% CI, −1.98% to −1.41%]; *P* = .005). This occurred to the greatest degree among low-income patients through the acquisition of Medicaid (APC, 11.0% [95% CI, 8.6%-13.3%]; *P* < .001). Implementation of the ACA was also associated with an increase in detection of stage I and II disease (APC, 4.0% [95% CI, 1.6%-6.3%]; *P* = .001) among low-income patients in expansion states.

**Conclusions and Relevance:**

Among patients with RCC, ACA implementation was associated with an increase in insurance coverage status in both expansion and nonexpansion states for all income groups, but to a greater degree in expansion states. The proportion of patients with localized disease increased among low-income patients in both states. These data suggest that ACA implementation is associated with earlier RCC detection among lower-income patients.

## Key Points

**Question:**

Was the Patient Protection and Affordable Care Act (ACA) associated with changes in insurance coverage and stage of diagnosis for patients with renal cell carcinoma (RCC), and were differences based on income?

**Findings:**

In this cohort study of 78 099 patients with RCC, the ACA was associated with increased insurance coverage through Medicaid for low-income patients and detection at an earlier stage of disease. Insurance coverage increased to a greater degree in states that expanded their Medicaid eligibility.

**Meaning:**

These findings suggest that the ACA was associated with significant increases in insurance coverage for lower-income patients and early diagnosis of RCC.

## Teaser

This cohort study tests the hypothesis that implementation of the Patient Protection and Affordable Care Act may be associated with increased access to care through expansion of insurance, which may vary based on income, among patients with renal cell carcinoma.

## Introduction

The Patient Protection and Affordable Care Act (ACA) is considered by many to be the most significant change in health care in the US since the passage of the Social Security Act Amendments of 1965 (Medicare and Medicaid Act).^1^ The ACA focuses on improving access to care by increasing health insurance coverage.^2^ Provisions of the ACA increased the availability of health insurance through several mechanisms, including elimination of preexisting conditions for insurance coverage denial, an employer mandate to offer health insurance, establishment of a marketplace for individuals to purchase insurance, penalty for not having insurance, and expansion of Medicaid eligibility.^1^

In this framework, states had an opportunity to opt out of Medicaid expansion. In nonexpansion states, individuals may qualify for Medicaid if they make 40% of the poverty threshold ($4996 gross income for a family of 1). In contrast, in an expansion state, individuals may qualify for Medicaid if they make 133% of the poverty threshold ($16 237 gross income for a family of 1).^3^ The main provisions of the ACA—including the individual mandate, employer mandate, preexisting conditions policy, and expansion of Medicaid eligibility—were implemented in 2014. It was hypothesized that owing to overall health policy changes of the ACA, individuals would increase access to insurance and therefore cancer care.^4,5^

Several studies have examined the effect of the ACA on cancer care, and the preponderance of data suggest that the ACA effectively increased insurance for individuals with a diagnosis of cancer.^4,6^ Although this topic has been explored in testicular and prostate cancer, it has yet to be addressed in renal cell carcinoma (RCC).^6,7,8^ We hypothesized that ACA implementation was associated with changes in insurance coverage status and stage at diagnosis for patients with RCC.

## Methods

### Patient Population

This study followed the Strengthening the Reporting of Observational Studies in Epidemiology (STROBE) reporting guideline for cohort studies. This was a retrospective analysis of patients aged 40 to 64 years diagnosed with RCC from January 1, 2010, to December 31, 2016, using the National Cancer Database (NCDB).^9,10^ The data were analyzed from July 1 to December 31, 2020. We restricted our cohort to cortical neoplasms (*International Classification of Diseases for Oncology, Third Edition*, histological codes 8255, 8260, 8263, 8270, 8290, 8312, 8314, 8316, 8317, and 8318 [clear cell carcinoma, cyst-associated RCC, oncocytic/chromophobe RCC, papillary RCC, and sarcomatoid RCC]). We used the *AJCC Cancer Staging Manual*, 7th edition, TNM staging system and stratified patients between those with advanced cancer (stage III and IV) and localized cancer (stage I and II) .^9^ We focused only on states that expanded Medicaid eligibility in 2014 and states that did not expand Medicaid coverage during the study period. We used 2014 to 2016 as the era after ACA implementation and 2010 to 2013 as the era before ACA implementation and included patients who had Medicaid, private insurance, or no insurance at time of diagnosis. Institutional review board approval and informed consent were not required because all data were deidentified and publicly available.

**Figure 1.**
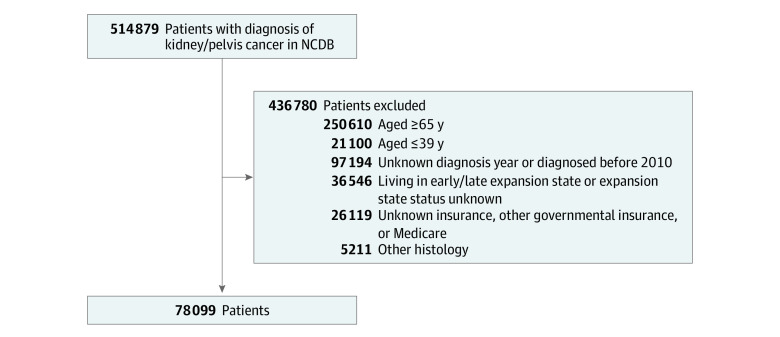
CONSORT Diagram NCDB indicates National Cancer Database.

Exclusion criteria included age 65 years or older (due to Medicare eligibility) and 39 years or younger (geographic data unavailable in the NCDB). To avoid bias, we excluded residents in states with early or late implementation of Medicaid expansion, because some states expanded Medicaid coverage before and after 2014. The assignment of patients into these groups is performed by the NCDB based on the patient’s address. The NCDB does not provide state-level data or address information about patients. We also excluded patients with coverage by other governmental insurance such as Tricare^11^ as well as insurance status unknown (Figure 1).

### Data Collection

Data were collected using the NCDB 2016 participant user file, which contains cases submitted to the Commission on Cancer’s NCDB. The NCDB is a joint program between the Commission on Cancer of the American College of Surgeons and the American Cancer Society. The database captures approximately 70% of all newly diagnosed cases of cancer in the US^10^ and provides patient-level data for analysis of cancer outcomes.

The NCDB provides 4 ranges of patient income: less than $40 227, $40 227 to $50 353, $50 354 to $63 332, and $63 333 or greater. Patients were stratified by median family income into low, middle, and high income. We converted patient incomes into federal poverty guidelines (FPG) applying poverty thresholds as described by Hsiang et al.^12^ We stratified patients into low-income (≤153% FPG), middle-income (154%-240% FPG), and high-income (≥241% FPG) groups. For racial designation, patients were grouped as White, Black, American Indian, Asian, other (multiracial), or unknown. For ethnicity, patients were grouped as Hispanic or non-Hispanic. These classifications were provided by the NCDB. Patients were classified as male or female. Patients were stratified into age groups at time of the diagnosis as 40 to 49, 50 to 59, and 60 to 64 years. Patients were stratified based on the educational attainment of their county as designated in the NCDB based on the percentage of inhabitants who live in a region with no high school degree. For comorbidity, patients were categorized into 2 groups based on Charlson comorbidity index score (score of 0 or score of ≥1).

### Statistical Analysis

The primary outcome was change in health insurance comparing expansion and nonexpansion states. Secondary outcomes included change in stage at diagnosis, difference in the rate of insurance change, and change in localized disease between expansion and nonexpansion states.

Patient demographic, clinical, and treatment characteristics were reported as categorical variables. Differences in patient demographics, socioeconomic factors, and disease stage between Medicaid expansion and nonexpansion groups were assessed with Pearson χ^2^ tests. Stage trend analysis was performed to assess for stage changes based on income status among patients living in expansion and nonexpansion states. Pearson χ^2^ test without continuity correction was used to assess whether or not there was significant change in number of patients with insurance and specific stages. Absolute percentage change (APC) was calculated for insurance status and stage migration. We performed the Cochran-Armitage test to evaluate linear trends in rates of insurance and the rates of localized disease.^13^

Difference-in-difference (DID) modeling was performed to examine differences in obtaining insurance and in the rate of being diagnosed with localized disease between expansion and nonexpansion states (eMethods in the Supplement).^14^ The parallel trend assumption was met for the insurance data by visual inspection. Univariable and multivariable linear regression was performed when time of intervention was defined as 2014 or later as year of diagnosis. The control population consisted of patients living in the nonexpansion states, and the treated population included patients living in the expansion states. The interaction term was defined as the time of intervention and state where a patient lived. In our multivariable model, we adjusted for the following variables: sex, age, race, ethnicity, income, educational attainment, comorbidity, and stage or insurance status when applicable. These variables were selected based on prior publication.^4,12^

Statistical analyses were performed using SPSS, version 27 (IBM Corporation) and R Studio, version 1.3.959 (R Program for Statistical Computing). Two-sided *P* < .05 was considered statistically significant.

## Results

After exclusions, 78 099 patients were included in the analysis (50 565 [64.7%] male and 27 534 [35.3%] female; mean [SD] age at diagnosis, 54.66 [6.46] years); of 77 066 with income data available, 16 333 (21.2%) had low incomes, 35 610 (46.2%) had middle incomes, and 25 123 (32.6%) had high incomes. Significantly higher proportions of low-income areas (10 637 of 41 833 [25.4%] vs 5696 of 35 233 [16.2%]; *P* < .001), areas with higher proportions of residents without a high school degree (≥17.6%, 11 619 of 41 887 [27.7%] vs 6744 of 35 307 [19.1%]; *P* < .001), and uninsured patients (4390 of 42 485 [10.3%] vs 1701 of 35 613 [4.8%]; *P* < .001) were noted in nonexpansion states (Table 1).

**Table 1.** Demographics and Disease Characteristics

Category	No. (%) of patients^a^		
	All	Nonexpansion states	Expansion states
Age at diagnosis, y			
40-49	18 497 (23.7)	10 136 (23.9)	8361 (23.5)
50-59	37 949 (48.6)	20 707 (48.7)	17 242 (48.4)
60-64	21 653 (27.7)	11 643 (27.4)	10 010 (28.1)
Sex			
Male	50 565 (64.7)	27 143 (63.9)	23 422 (65.8)
Female	27 534 (35.3)	15 343 (36.1)	12 191 (34.2)
Ethnicity			
Hispanic	6224 (8.2)	3821 (9.2)	2403 (7.0)
Non-Hispanic	69 798 (91.8)	37 647 (90.8)	32 151 (93.0)
Race			
White	64 648 (83.6)	35 004 (83.0)	29 644 (84.4)
Black	9883 (12.8)	6124 (14.5)	3759 (10.7)
American Indian	396 (0.5)	221 (0.5)	175 (0.5)
Asian	1414 (1.8)	362 (0.9)	1052 (3.0)
Other^b^	960 (1.2)	475 (1.1)	485 (1.4)
CCI score			
0	21 432 (27.4)	11 843 (27.9)	9589 (26.9)
≥1	56 667 (72.6)	30 643 (72.1)	26 024 (73.1)
Median income			
Low	16 333 (21.2)	10 637 (25.4)	5696 (16.2)
Middle	35 610 (46.2)	20 231 (48.4)	15 379 (43.6)
High	25 123 (32.6)	10 965 (26.2)	14 158 (40.2)
AJCC stage			
I	44 257 (66.6)	23 997 (65.7)	20 260 (67.7)
II	7946 (12.0)	4477 (12.3)	3469 (11.6)
III	4958 (7.5)	2806 (7.7)	2152 (7.2)
IV	9285 (14.0)	5227 (14.3)	4058 (13.6)
Insurance status			
Uninsured	6091 (7.8)	4390 (10.3)	1701 (4.8)
Medicaid	9114 (11.7)	3839 (9.0)	5275 (14.8)
Private	62 893 (80.5)	34 256 (80.6)	28 637 (80.4)
No high school degree, %			
<6.3	16 956 (22.0)	7812 (18.7)	9144 (25.9)
6.3-10.8	20 745 (26.9)	10 501 (25.1)	10 244 (29.0)
10.9-17.5	21 130 (27.4)	11 955 (28.5)	9175 (26.0)
≥17.6	18 363 (23.8)	11 619 (27.7)	6744 (19.1)

Abbreviations: AJCC, American Joint Committee on Cancer; CCI, Charlson comorbidity index.

^a^ Percentages have been rounded and may not total 100.

^b^ Includes multiracial patients and patients of unknown race.

Before ACA implementation, greater proportions of uninsured patients lived in nonexpansion states (2602 of 23 008 [11.3%]) compared with expansion states (1281 of 19 351 [6.6%]; *P* < .001) (Table 2). After ACA implementation, proportions of uninsured patients declined in both nonexpansion (1788 of 19 477 [9.2%]) and expansion (420 of 16 262 [2.6%]) states (*P* < .001). In expansion states, insurance coverage increased secondary to Medicaid enrollment; this occurred to the greatest degree among low-income patients through the acquisition of Medicaid (APC, 11.0% [95% CI, 8.6%-13.3%]; *P* < .001). More specifically, when comparing before and after the ACA, the number of patients with Medicaid was 704 of 3057 (23.0%) vs 897 of 2639 (34.0%), respectively, for low income; 1036 of 8255 (12.5%) vs 1475 of 8809 (16.7%), respectively, for middle income; and 469 of 7796 (6.0%) vs 636 of 6362 (10.0%), respectively, for high income (all *P* < .001). In nonexpansion states, the proportion of patients with Medicaid was stable overall (pre-ACA, 2110 of 23 008 [9.2%]; post-ACA, 1729 of 19 477 [8.9%]; *P* = .37) and across income groups, although there was a significant increase in the proportion of patients obtaining private insurance overall (APC, 2.4% [95% CI, 1.6%-3.1%]; *P* < .001) and across income groups (low income, 3954 of 5771 [68.5%] vs 3486 of 4865 [71.7%]; middle income, 8773 of 10 949 [80.1%] vs 7643 of 9282 [82.3%]; high income, 5287 of 5929 [89.2%] vs 4588 of 5036 [91.1%]; *P* ≤ .001 for all).

**Table 2.** Insurance Trend Analysis by Expansion Status Stratified by Income

Insurance status by income level	Nonexpansion states				Expansion states			
	ACA implementation, No. (%)^a^		APC (95% CI), %	*P* value	ACA implementation, No. (%)^a^		APC (95% CI), %	*P* value
	Before	After			Before	After		
All								
Uninsured	2602 (11.3)	1788 (9.2)	−2.1 (−1.5 to −2.7)	<.001	1281 (6.6)	420 (2.6)	−4.0 (−2.5 to 6.6)	<.001
Medicaid	2110 (9.2)	1729 (8.9)	−0.3 (−0.8 to 0.2)	.37	2238 (11.6)	3037 (18.7)	7.7 (6.3 to 7.8)	<.001
Private	18 296 (79.5)	15 960 (81.9)	2.4 (1.6 to 3.1)	<.001	15 832 (81.8)	12 805 (78.7)	−3.1 (−3.9 to −2.2)	<.001
Low								
Uninsured	929 (16.1)	678 (13.9)	−2.2 (−3.5 to 0.8)	.001	373 (12.2)	99 (3.8)	−8.4 (−9.8 to −7.0)	<.001
Medicaid	888 (15.4)	701 (14.4)	1.0 (−2.3 to 0.3)	.16	704 (23.0)	897 (34.0)	11.0 (8.6 to 13.3)	<.001
Private	3954 (68.5)	3486 (71.7)	3.2 (1.3 to 4.8)	<.001	1980 (64.8)	1643 (62.3)	−2.5 (−3.4 to 1.5)	.049
Middle								
Uninsured	1222 (11.2)	826 (8.9)	−2.3 (−3.0 to −1.4)	<.001	603 (7.3)	210 (2.4)	−5.0 (−5.5 to −4.2)	<.001
Medicaid	954 (8.7)	813 (8.8)	0.1 (−0.7 to 0.8)	.91	1036 (12.5)	1475 (16.7)	4.2 (3.1 to 5.2)	<.001
Private	8773 (80.1)	7643 (82.3)	2.2 (1.1 to 3.0)	<.001	6616 (80.1)	7124 (80.9)	0.7 (−0.4 to 1.9)	.23
High								
Uninsured	410 (6.9)	258 (5.1)	−1.8 (−2.6 to −0.8)	<.001	283 (3.6)	108 (1.7)	−1.9 (−2.4 to −1.4)	<.001
Medicaid	232 (3.9)	190 (3.8)	−0.1 (−0.8 to 0.5)	.70	469 (6.0)	636 (10.0)	4.0 (3.1 to 4.8)	<.001
Private	5287 (89.2)	4588 (91.1)	1.9 (1.0 to 3.2)	.001	7044 (90.4)	5618 (88.3)	−2.1 (−3.0 to −1.0)	<.001

Abbreviations: ACA, Patient Protection and Affordable Care Act; APC, absolute percentage change.

^a^ Percentages have been rounded and may not total 100.

Comparing the periods before and after ACA implementation, similar increases occurred in the proportion of stage I and II diagnoses in nonexpansion states (APC, 1.5% [95% CI, 0.6%-2.3%]; *P* < .001) and expansion states (APC, 1.1% [95% CI, 0.1%-2.0%]; *P* = .02) (Table 3). When broken down by income status, we noted the highest increase in downward stage migration in low-income patients (APC, 4.0%; *P* < .001) and middle-income patients (APC, 1.6%; *P* = .02) in expansion states.

**Table 3.** Cancer Stage Trend Analysis by Expansion Status Stratified by Income

Cancer stage by income level	Nonexpansion states				Expansion states			
	ACA implementation, No. (%)^a^		APC (95% CI), %	*P* value	ACA implementation, No. (%)^a^		APC (95% CI), %	*P* value
	Before	After			Before	After		
All								
I and II	15 298 (77.3)	13 176 (78.8)	1.5 (0.6 to 2.3)	<.001	12 788 (78.8)	10 951 (79.9)	1.1 (0.1 to 2.0)	.02
III and IV	4487 (22.7)	3546 (21.2)	−1.5 (−2.3 to −0.6)		3447 (21.2)	2763 (20.1)	−1.1 (−2.0 to −0.1)	
Low								
I and II	3839 (76.5)	3240 (77.7)	1.2 (−0.5 to 2.8)	.20	1934 (76.7)	1807 (80.7)	4.0 (1.6 to 6.3)	.001
III and IV	1177 (23.5)	932 (22.3)	−1.2 (−2.8 to 0.5)		589 (23.3)	433 (19.3)	−4.0 (−6.3 to −1.6)	
Middle								
I and II	7241 (76.8)	6283 (78.2)	1.4 (0.2 to 2.6)	.02	5321 (77.7)	4793 (79.3)	1.6 (0.2 to 3.0)	.02
III and IV	2189 (23.2)	1747 (21.8)	−1.4 (−0.2 to −2.6)		1528 (22.3)	1248 (20.7)	−1.6 (−3.0 to −0.2)	
High								
I and II	3982 (79.3)	3453 (80.9)	1.6 (0.02 to 3.2)	.06	5375 (80.7)	4252 (80.0)	−0.7 (−2.2 to 0.6)	.29
III and IV	1038 (20.7)	814 (19.1)	−1.6 (−3.2 to 0.02)		1282 (19.3)	1066 (20.0)	0.7 (−0.6 to 2.2)	

Abbreviations: ACA, Patient Protection and Affordable Care Act; APC, absolute percentage change.

^a^ Percentages have been rounded and may not total 100.

**Figure 2.**
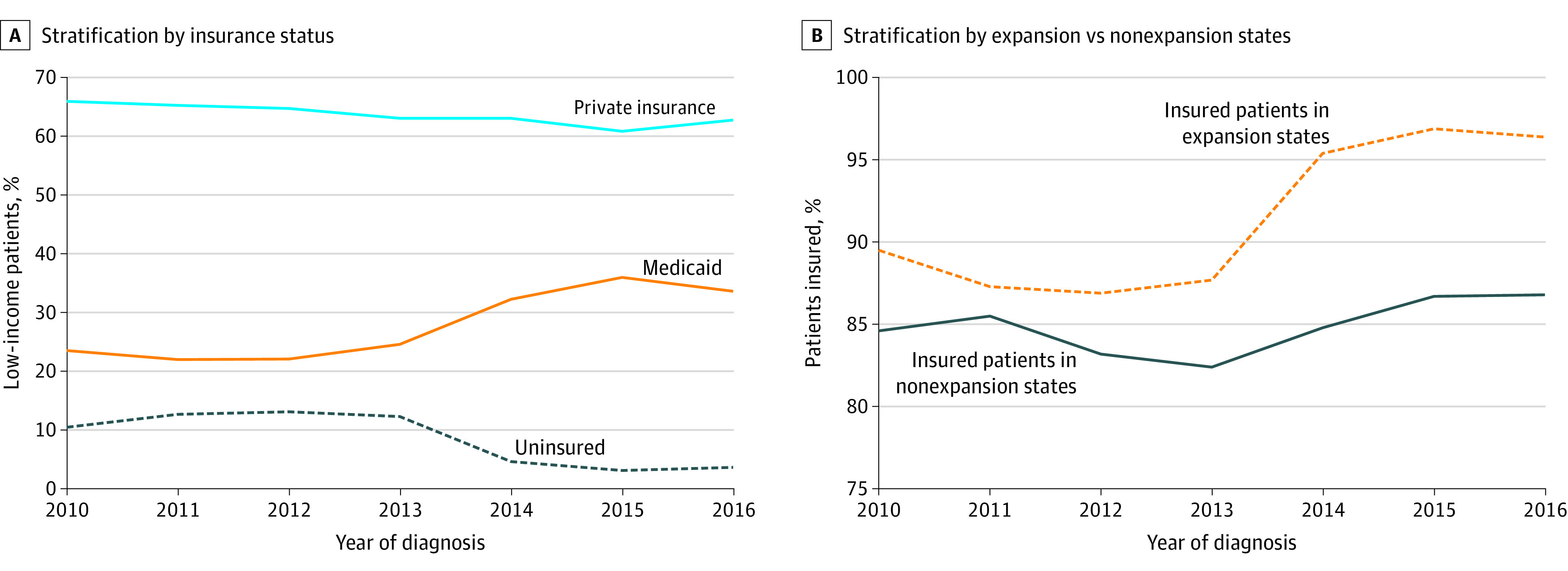
Insurance Trend Analysis for Low-Income Patients Data are stratified by insurance status and by expansion and nonexpansion states.

When evaluating low-income patients in expansion states using the Cochrane-Armitage trend test, we noted important changes. Comparing rates for different forms of insurance for 2013 to 2016, private insurance remained stable (63.1% to 62.8%; *P* = .84), whereas an increase was noted in Medicaid (24.6% to 33.6%; *P* < .001) and the overall rate of uninsured patients declined (12.3% to 3.6%; *P* < .001) (Figure 2A). When analyzing low-income patients, the percentage of insured patients increased from 89.5% to 96.4% in expansion states, and from 84.6% to 86.8% in nonexpansion states (Figure 2B).

When evaluating stage trends for low-income patients and comparing nonexpansion with expansion states using the Cochrane-Armitage trend test, we also noted important differences. Before ACA implementation, rates from 2010 to 2013 of stages I and II disease in low-income patients remained stable in expansion states (75.0% to 77.2%; *P* = .33) and nonexpansion states (76.8% to 76.1%; *P* = .28). After ACA implementation, proportions of low-income patients with stage I and II cancer increased from 2013 to 2016, with higher magnitude increases noted in expansion states (77.2% to 81.3%; *P* = .003) compared with nonexpansion states (76.1% to 78.3%; *P* = .02) (eFigure in the Supplement).

We performed DID analysis to compare changes in difference in reduction of being uninsured for expansion vs nonexpansion states and demonstrated that after ACA implementation, expansion states had a greater reduction of the rate of uninsured patients compared with nonexpansion states (−1.14% [95% CI, −1.98% to −1.41%]; *P* = .005) (eTable 1 in the Supplement). The adjusted reduction in the uninsured rate comparing expansion and nonexpansion states was −4.39% (95% CI, −6.71% to −2.08%; *P* < .001) for low-income patients and −1.52% (95% CI, −2.75% to −0.28%; *P* = .02) for middle-income patients. DID analysis comparing changes in difference in the increase of the proportion of localized disease for expansion vs nonexpansion states revealed no significant difference between the increase in the proportion of patients with stage I and II diagnosis among low-income patients in expansion and nonexpansion states (2.69% [95% CI, −0.23% to 5.63%]; *P* = .07) (eTable 2 in the Supplement). In addition, there were no significant differences in rate of localized disease between expansion and nonexpansion states for middle-income (0.80% [95% CI, −1.10% to 2.70%]; *P* = .41) and high-income (−1.99% [95% CI, − 4.18% to 0.18%]; *P* = .07) groups.

## Discussion

In this cohort study, we present—to our knowledge—the first analysis of the association of the ACA with outcomes in patients with RCC. Our findings suggest that ACA implementation was associated with improved access to care through insurance and was also associated with the diagnosis of RCC at earlier stages in low-income patients. We observed that the proportion of patients diagnosed with RCC who had insurance increased in both expansion and nonexpansion states through either Medicaid or private insurance, the greatest associations of which were seen in low-income patients through acquisition of Medicaid in expansion states. Furthermore, the proportion of patients with localized disease increased after ACA implementation. This was seen to the greatest degree in low-income patients living in Medicaid expansion states. Our findings suggest that ACA implementation is associated with detection of RCC at early stages among lower-income patients.

The provisions of the ACA provided multiple pathways for individuals to gain health insurance beyond the expansion of Medicaid eligibility. Although many states expanded patient eligibility for Medicaid, there remained gaps in coverage. Employer mandates aimed to alleviate this gap, as did subsidies for private insurance for patients with incomes at 138% to 400% of the poverty line.^15^ The myriad pathways to gain insurance were ultimately successful in expanding health insurance for patients. Multiple studies have shown that uninsured rates in low-income groups living in expansion states significantly decreased after ACA implementation.^16,17^ Hsiang et al^12^ evaluated 30 842 patients with testicular cancer using data from the NCDB from 2010 to 2015 (70.2% aged <39 years) and demonstrated that the largest increases in insurance coverage were among low-income patients through Medicaid with an increase in APC of 14.5% (95% CI, 7.2%-21.8%). Similar to our findings, the authors noted that in nonexpansion states, patients were more likely to gain coverage through private insurance (APC, 6.8%); however, they found no differences between rates of uninsured between expansion and nonexpansion states overall and when stratified by income in their adjusted DID analysis. In contrast, Weiner et al^18^ studied 41 329 patients with testicular cancer from the NCDB (median age, 47 years) from 2007 to 2016 and noted a greater decrease in the uninsured rate (DID, −4.20%; *P* = .02) in expansion compared with nonexpansion states. These findings are similar to those of our study, because we found overall higher reduction in noninsurance rates in expansion states (DID, −1.14%; *P* = .005) particularly in low-income (DID, −4.39%; *P* < .001) and middle-income (DID, −1.52%; *P* = .02) patients. Our results likely mirror those of Weiner et al^18^ because of the similarity in the age of our cohorts, with approximately 70% of our cohort aged 40 to 59 years. The effect of the ACA based on age has not been extensively studied in a direct manner. The positive association of the ACA with insurance rates among low-income patients was notable in our cohort as well as that of Weiner et al,^18^ as opposed to that of Hsiang et al,^12^ and suggest that older groups may derive more benefit.

It should be noted that we identified high-income individuals as being more likely to acquire Medicaid after eligibility expansion in Medicaid expansion states. This is likely secondary to the holistic approach for determining Medicaid eligibility. Medicaid eligibility is based on family size, assets, and expenses, such as rent. Moreover, high income was defined as a gross income of greater than $46 000. It is likely that a substantial portion of patients with incomes equal to or greater than $46 000 have a gross income within 133% of the federal poverty line ($16 237 gross income for family of 1) when adjusted for expenses and family size. Our group is among the first to identify this association between ACA implementation and Medicaid eligibility.^15^

We noted greater proportions of patients presenting with localized disease after implementation of the ACA in both low- and middle-income groups living in expansion states (APC, 4.0% [*P* = .001] and 1.6% [*P* = .02], respectively). This is further demonstrated in trend analysis where the percentage of localized disease increased from 75.0% in 2010 to 81.3% in 2016 (*P* < .001). Based on our findings, the implementation of the ACA as a whole was associated with an increase in the proportion of patients being diagnosed with localized disease. Nonetheless, the effect of ACA implementation on stage trends in genitourinary malignant neoplasms is conflicting, depending on the years analyzed. Han et al^3^ analyzed 2 471 154 patients using cancer registries of 40 individual states for 1 year after ACA implementation (2014) and noted that stage at diagnosis shifted slightly to an earlier stage for most cancer types, but for kidney/renal pelvis neoplasms (18-64 years of age), there was no significant difference between expansion and nonexpansion states (−0.9%; *P* = .32). Francis et al^17^ used the NCDB to examine the effect of the ACA on testicular cancer, noting worsening stage migration for testicular cancer (8.9% vs 31.9%; *P* < .01). Differences between our findings and those of Han et al^3^ and Francis et al^17^ may be owing to several factors. First, we excluded early and late expansion states and sought a direct comparison of expansion and nonexpansion states during a defined period where trends may be ascertained; therefore, unlike Han et al,^3^ we examined a longer period and were able to determine trends for RCC. Second, Francis et al^17^ defined the post-ACA period as 2011 to 2013, when most states had not implemented ACA, and there was no analysis or stratification based on whether or not a state had expanded Medicaid eligibility.

### Limitations

Our analysis has potential limitations. First, retrospective studies are subject to inherent biases of analytic design. Second, the nature of findings with a total of 3 years after ACA implementation are short term, and more follow-up on patients diagnosed after ACA implementation is necessary to ascertain whether changes in insurance rates and coverage and rate of localized disease will evolve further. Third, despite the size of the cohort, some minorities may be undercaptured in population-based cancer registry data sets, and therefore results and conclusions regarding outcomes in minority groups may not be representative.^19^ Fourth, we do not have state-level data, because these are unavailable in the NCDB and therefore may mask differences between states. Thus, income level by state may have confounded the results of this study. However, patients are categorized in the database as living in an expansion state or a nonexpansion state; thus, our results are generalizable to association with the ACA. Fifth, owing to the limitations of the data set, we could not treat income as a continuous variable and thus could not determine a maximal cutoff point for when the association with the ACA stopped. Finally, the NCDB may overrepresent urban and academic medical centers and may not adequately characterize the effect of the ACA on rural centers.

## Conclusions

The findings of this cohort study show that after ACA implementation, insurance coverage status increased for both expansion and nonexpansion states for all patients, with the most pronounced changes occurring among low-income patients living in expansion states. Implementation of the ACA was associated with increased rates of insurance and early diagnosis of RCC at earlier stages in low-income patients. Our hypothesis-forming findings suggest that the implementation of the ACA has been salutary, especially for low-income patients diagnosed with RCC, and call for further longitudinal follow-up and investigation into the potential associations between the ACA and disparities in health care and outcomes.

## Notes

Supplement. **eTable 1.** Insurance Trend Analysis Stratified by Income Comparing Nonexpansion vs Expansion States **eTable 2.** Stage Trend Analysis Stratified by Income Comparing Nonexpansion vs Expansion States **eFigure.** Stage Trend Analysis of Localized Disease for Low-Income Patients in Expansion and Nonexpansion States **eMethods.** Differences-in-Differences Analysis
