# Supplementary material for: Evaluation of Insurance Coverage and Cancer Stage at Diagnosis Among Low-Income Adults With Renal Cell Carcinoma After Passage of the Patient Protection and Affordable Care Act
Source: JAMA Netw Open. 2021 Jul 16;4(7):e2116267. doi: 10.1001/jamanetworkopen.2021.16267 (PMC8285737; doi:10.1001/jamanetworkopen.2021.16267)
Supplement: Supplement. — eTable 1. Insurance Trend Analysis Stratified by Income Comparing Nonexpansion vs Expansion States eTable 2. Stage Trend Analysis Stratified by Income Comparing Nonexpansion vs Expansion States eFigure. Stage Trend Analysis of Localized Disease for Low-Income Patients in Expansion and Nonexpansion States eMethods. Differences-in-Differences Analysis [file jamanetwopen-e2116267-s001.pdf]

Supplementary Online Content

Javier-DesLoges JF, Yuan J, Soliman S, et al. Evaluation of insurance coverage and cancer stage at diagnosis among low-income adults with renal cell carcinoma after passage of the Patient Protection and Affordable Care Act. *JAMA Netw Open*. 2021;4(7):e2116267. doi:10.1001/jamanetworkopen.2021.16267

eTable 1. Insurance Trend Analysis Stratified by Income Comparing Nonexpansion vs Expansion States
eTable 2. Stage Trend Analysis Stratified by Income Comparing Nonexpansion vs Expansion States
eFigure. Stage Trend Analysis of Localized Disease for Low-Income Patients in Expansion and Nonexpansion States
eMethods. Differences-in-Differences Analysis

This supplementary material has been provided by the authors to give readers additional information about their work.

© 2021 Javier-DesLoges JF et al. *JAMA Network Open*.

eTable 1. Insurance Trend Analysis Stratified by Income Comparing Nonexpansion vs Expansion States

	Unadjusted DID	95% CI	p value	Adjusted DID	95% CI	p value
All income						
Uninsured vs. Insured	-1.90%	-2.66% - 1.15%	<0.001	-1.14%	- 1.98% – -1.41%	0.005
Low Income						
Uninsured vs. Insured	-6.28%	-8.42% - -4.15%	<0.001	-4.39%	-6.71% - -2.08%	0.001
Middle Income						
Uninsured vs. Insured	-2.09%	-3.23% - -0.95%	<0.001	-1.52%	-2.75% - -0.28%	0.016
High Income						
Uninsured vs. Insured	+0.37	-0.88% - 1.63%	0.558	+0.41%	-0.66% - 1.50%	0.449

Abbreviations: DID, Difference-In-Difference; CI, Confidence Interval

© 2021 Javier-DesLoges JF et al. *JAMA Network Open*.

eTable 2. Stage Trend Analysis Stratified by Income Comparing Nonexpansion vs Expansion States

	Unadjusted DID	95% CI	p value	Adjusted DID	95% CI	p value
All income						
Localized vs. Advanced	-0.37%	-1.63% - -0.88%	0.558	+0.10%	-1.19%- 1.35%	0.901
Low Income						
Localized vs. Advanced	+2.88%	0.00%-5.81%	0.053	+2.69%	-0.23%- 5.63%	0.071
Middle Income						
Localized vs. Advanced	+0.19%	-1.69%- 2.08%	0.841	+0.80%	-1.10% - 2.70%	0.410
High Income						
Localized vs. Advanced	-2.38%	-4.55%- - 0.21%	0.031	-1.99%	- 4.18%- 0.18%	0.073

Abbreviations: DID, Difference-In-Difference; CI, Confidence Interval

© 2021 Javier-DesLoges JF et al. *JAMA Network Open*.

eFigure. Stage Trend Analysis of Localized Disease for Low-Income Patients in Expansion and Nonexpansion States

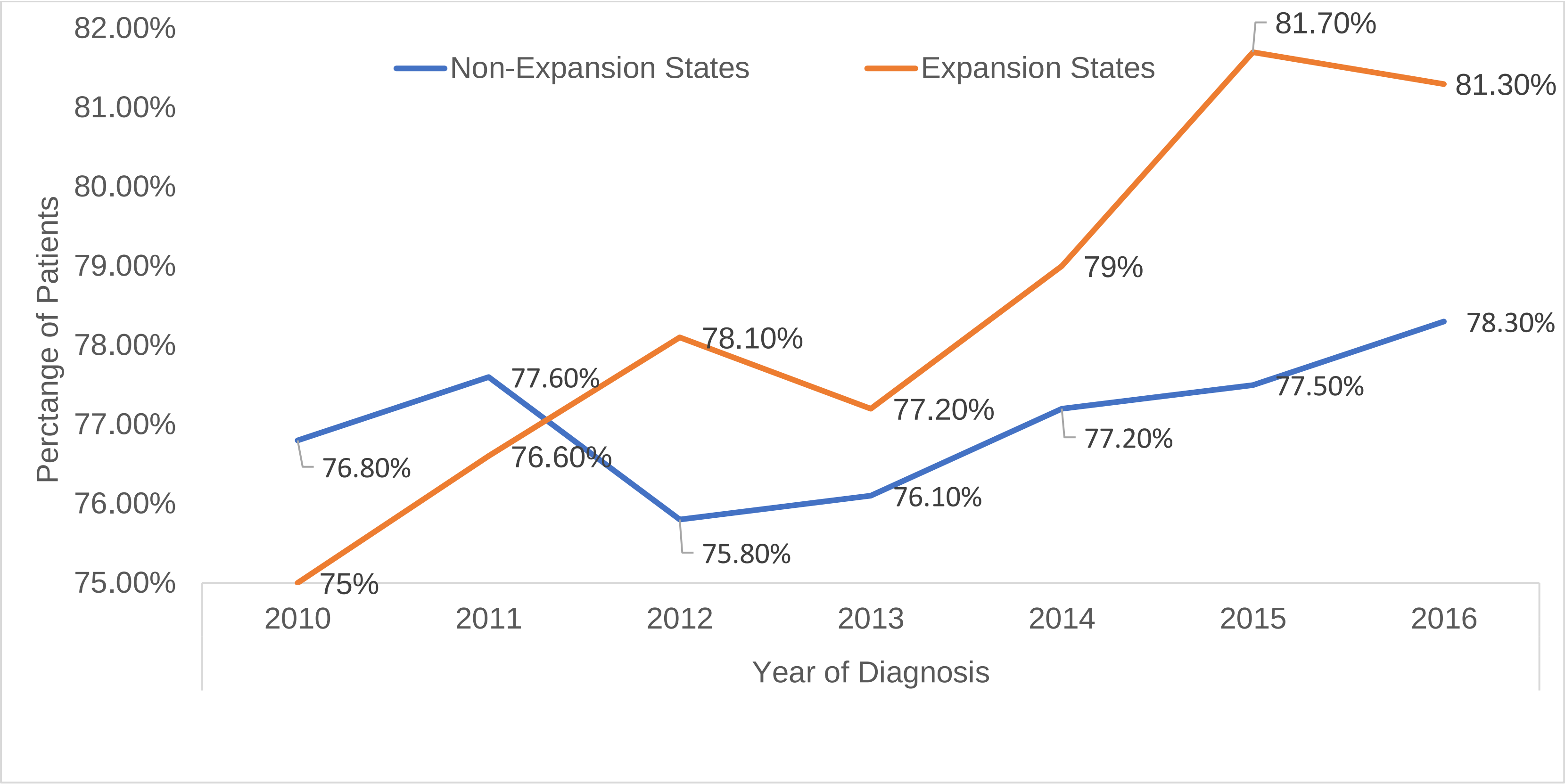

© 2021 Javier-DesLoges JF et al. *JAMA Network Open*.

eMethods. Differences-in-Differences Analysis

We performed Difference-in-Difference (DID) modeling to examine the interaction between the time of implementation of the ACA and the treated population. Time was defined time as greater than or equal to the year of diagnosis of 2014 ($\beta_2 Time$). The control population were patients living in non-expansion states and the treated population were patients living in the expansion states ($\beta_1 Expansion$). We performed an unadjusted (Equation 1 and 3) and adjusted DID analysis adjusting for sex, age, race, ethnicity, income, education, stage and, comorbidity (Equation 2 and 4).

In Equation 1 and 2, insurance was the dependent variable. In Equation 3 and 4, stage of disease was the dependent variable. We performed this analysis for patients of all incomes and stratified patients based on income status and performed a secondary analysis.

Equation 1:

$$Insurance\ (Y) = \beta_0 + \beta_1 Expansion + \beta_2 Time + \beta_3 (Expansion \times Time) + \varepsilon$$

Equation 2:

$$Insurance\ (Y) = \beta_0 + \beta_1 Expansion + \beta_2 Time + \beta_3 (Expansion \times Time) + \beta_4 Sex + \beta_5 Age + \beta_6 Race + \beta_7 Ethnicity + \beta_8 Income + \beta_9 Comorbidity + \beta_{10} Stage + \beta_{11}\ Education + \varepsilon$$

Equation 3:

$$Stage\ (Y) = \beta_0 + \beta_1 Expansion + \beta_2 Time + \beta_3 (Expansion \times Time) + \varepsilon$$

Equation 4:

$$Stage\ (Y) = \beta_0 + \beta_1 Expansion + \beta_2 Time + \beta_3 (Expansion \times Time) + \beta_4 Sex + \beta_5 Age + \beta_6 Race + \beta_7 Ethnicity + \beta_8 Income + \beta_9 Comorbidity + \beta_{10}\ Education + \varepsilon$$

© 2021 Javier-DesLoges JF et al. *JAMA Network Open*.
